# Alpha-Lipoic Acid and Glucose Metabolism: A Comprehensive Update on Biochemical and Therapeutic Features

**DOI:** 10.3390/nu15010018

**Published:** 2022-12-21

**Authors:** Umberto Capece, Simona Moffa, Ilaria Improta, Gianfranco Di Giuseppe, Enrico Celestino Nista, Chiara M. A. Cefalo, Francesca Cinti, Alfredo Pontecorvi, Antonio Gasbarrini, Andrea Giaccari, Teresa Mezza

**Affiliations:** 1 Endocrinology and Diabetology Unit, Fondazione Policlinico Universitario Agostino Gemelli IRCCS, 00168 Rome, Italy; 2 Department of Translational Medicine and Surgery, Università Cattolica del Sacro Cuore, 00168 Rome, Italy; 3 Digestive Disease Center, Fondazione Policlinico Universitario Agostino Gemelli IRCCS, 00168 Rome, Italy

**Keywords:** alpha-lipoic, glucose metabolism, diabetes prevention, complications

## Abstract

Alpha-lipoic acid (ALA) is a natural compound with antioxidant and pro-oxidant properties which has effects on the regulation of insulin sensitivity and insulin secretion. ALA is widely prescribed in patients with diabetic polyneuropathy due to its positive effects on nerve conduction and alleviation of symptoms. It is, moreover, also prescribed in other insulin resistance conditions such as metabolic syndrome (SM), polycystic ovary syndrome (PCOS) and obesity. However, several cases of Insulin Autoimmune Syndrome (IAS) have been reported in subjects taking ALA. The aim of the present review is to describe the main chemical and biological functions of ALA in glucose metabolism, focusing on its antioxidant activity, its role in modulating insulin sensitivity and secretion and in symptomatic peripheral diabetic polyneuropathy. We also provide a potential explanation for increased risk for the development of IAS.

## 1. Introduction

Alpha-lipoic Acid (ALA) is a natural compound with diverse biochemical functions, especially in its reduced form dihydro-lipoic acid (DHLA). It acts as a metal chelator, regenerates endogenous antioxidants such as vitamins C and E and is a modulator of the signalling transduction of several pathways [1]. Many studies have indicated its potential role in the regulation of glucose metabolism, highlighting its effects on insulin sensitivity [2], insulin secretion [3], the reduction of circulating lipid levels [4] and the increase of nitric oxygen [5]. Further, ALA also seems to play a role in improving peripheral diabetic polyneuropathy [6]. It is, therefore, widely prescribed in both type 1 and type 2 diabetes (T1D; T2D) [7,8] diabetic neuropathy and in other insulin resistance conditions such as metabolic syndrome (MS), polycystic ovary syndrome (PCOS) and obesity [9,10,11].

However, numerous reports have been published in the last 15 years describing cases of Insulin Autoimmune Syndrome (IAS) in genetically predisposed subjects taking ALA. The first case was reported in Japan [12] and followed by reports from other countries, including Italy [13,14]. Our group has also described two new cases of IAS triggered by ALA and associated with HLA-DRB1*04:03, in two women [15].

Here we aim to review the main chemical and biological functions of ALA in glucose metabolism, focusing on its antioxidant activity, its role in modulating insulin sensitivity and secretion and in improving symptomatic peripheral diabetic polyneuropathy.

## 2. Chemistry and Metabolism of ALA

Alpha-lipoic acid, or 1,2 dithiolane-3-pentanoic acid or thioctic acid, is an endogenous cofactor of important enzyme complexes necessary to produce energy. Initially described as a vitamin used for bacterial growth, it was isolated for the first time in 1951 by the biochemist Lester Reed from a liver sample [16].

The term alpha-lipoic acid derives from the fact that it is soluble in fats. Its molecule consists of two atoms of sulphur and eight of carbon. It is amphiphilic and due to this property is readily taken up in tissues, including the nervous system, as it can cross the blood-brain central barrier [17,18]. It has an asymmetric carbon atom with two optical isomers, the dextrorotatory (R-ALA or +ALA) and the levo-rotatory (S-ALA or—ALA). R-ALA form is naturally available and can be found free or conjugated to lysine residues, making it an essential cofactor, while synthetic ALA is a racemic mixture of +ALA and—ALA (+/− ALA). The oxidized and reduced form (ALA/DHLA) works as a powerful antioxidant inactivating free radicals and reactive oxygen species (ROS) and enhancing the activity of other endogenous antioxidants (vitamin C, E, glutathione) [18,19].

Although its biosynthesis pathway is not yet entirely clear, there is evidence of ALA synthesis in the mitochondria of mammalian cells [20]. ALA synthesis starts from a fatty acid of eight carbon atoms and cysteine [21,22] probably in the liver, which also seems to be responsible for its catabolism [22].

ALA is found in high concentrations in both animal and plant sources, although the highest content is found in animal tissues with a high metabolic activity, such as the heart, while muscle tissue has lower concentrations [23]. Good animal sources of ALA are pork and calf meat, with the highest amounts found in heart, liver and kidneys. Good plant sources are spinach, broccoli, tomatoes, peas, brussels sprouts and rice bran (in descending order of content) [17]. However, in foods, the R enantiomer of alpha-lipoic acid is often covalently linked to the lysine residues of proteins, which reduces its systemic bioavailability [23].

## 3. ALA: Cellular Energy and Oxidative Stress

ALA is both an anti-oxidant and a pro-oxidant agent [24,25], inactivating free radicals and reactive oxygen species and modulating several signalling cascades involved in aging. ALA has diverse functions which can vary in relation to other compounds present in the cell, e.g., ALA can stimulate glucose uptake especially in muscles and adipocytes.

In describing the properties of this molecule, we will start by analysing the basic biochemical ones and then move on to those involved in glucose metabolism.

ALA is a cofactor of the pyruvate-dehydrogenase complex, which converts pyruvate obtained from glycolysis to acetyl-CoA. Lipoic acid in its oxidized form (ALA/lipoate) is first reduced (DHLA) and then oxidized again [26]. Through this biochemical reaction the pyruvate obtained from glycolysis can, by transforming into acetyl-CoA, start the Krebs cycle, which promotes the production of ATP and therefore the energy necessary for the cell to perform its functions. It has therefore been hypothesized that ALA could reduce the Warburg effect in cancer cells by stimulating the transformation of pyruvate into acetyl-CoA, rather than lactate, through the cofactor activity of the enzyme pyruvate-dehydrogenase (PDH) [27].

ALA acts as both a direct and indirect antioxidant: it is a scavenger of hydroxyl radicals, hypochlorous acid, singlet oxygen and peroxyl radicals [19,27]. It also increases the intracellular amount of glutathione and ascorbate [25,28], thus indirectly increasing the antioxidant ability of the cell and chelates iron and copper [24]. There is also some evidence that ALA can reduce the advanced glycation end products in neuronal cells with promising results for the treatment of Alzheimer’s disease [27].

ALA’s effects on cellular metabolism and oxidative stress probably have several systemic implications for both glucose and lipid metabolism. In the following sections we will discuss in depth how these properties can interact with glucose metabolism. We will first analyse ALA’s effects on insulin sensitivity and secretion, then discuss related diseases such as insulin resistance and diabetes. With regard to lipids, ALA seems to reduce both total cholesterol and triglycerides in animal models [4], suggesting a possible role also in the treatment of dyslipidaemias [29].

## 4. ALA and Glucose Metabolism

### 4.1. Effect of ALA on Glucose Uptake

Several studies over the past years have demonstrated that both ALA and DHLA can increase glucose uptake [2,30,31]. In particular, Eason et al. demonstrated that glucose uptake increased by 300% when muscles of ob/ob mice, a model of severe insulin resistance, were incubated with ALA alone, while no changes occurred when muscles were incubated only with insulin, thus demonstrating the effect of ALA on insulin resistance [30].

Further studies have confirmed this effect and several molecular pathways have been explored. One of these pathways includes the activation of the insulin receptor cascade. Treatment with R (+) alpha-lipoic acid stimulates the activity of PI3K and the phosphorylation of insulin receptor substrate- 1 (IRS-1) in 3T3-L1 adipocytes [31]. The phosphorylation of the substrate of the insulin receptor involves the activation of further intracellular mediators and subsequently the translocation of GLUT4 [31]. Diesel et al. revealed a direct binding site for alpha-lipoic acid at the tyrosine kinase domain of the insulin receptor in hepatocytes, suggesting a stabilizing function in loop A that is involved in ATP binding [32]. Thus, ALA can be considered an insulin-mimetic agent [1], since the bond of insulin receptor (IR) and IRS-1 phosphorylation could subsequently lead to GLUT4 translocation and increased glucose uptake. However, there are some controversies over ALA’s action on insulin signalling. In a preadipocyte cell culture exposed to ALA [33], Akt is phosphorylated within 30 min while the insulin receptor IR and the IRS-1 are not phosphorylated. Other research findings in adipocyte cell cultures demonstrate that IR is indeed phosphorylated but this is specific to ALA, the reduced form of the compound and not to DHLA [34].

Konrad et al. described two different mechanisms through which ALA induces glucose uptake: ALA induces PI3K and AKT phosphorylation, which determine GLUT4 translocation and it also increases p38 MAPK activity, which determines GLUT4 activation [2]. Therefore, GLUT4 activity seems to be a common element to several hypotheses since the activation of the insulin receptor cascade would also secondarily determine an increase in glucose uptake by GLUT4.

Furthermore, ALA has different effects on AMPK expression in peripheral tissues such as skeletal muscles and hypothalamus. A three-day ALA treatment increases glucose uptake, evaluated by euglycemic hyper-insulinemic clamp, reduces plasma lactate and increases α-2 AMPK activity in the skeletal muscle [35] while decreasing AMPK activity in the hypothalamus [36].

AMPK is a cellular fuel sensor whose activation is associated with ATP production through fatty acid oxidation. Moreover, it can induce the translocation of GLUT4 to the cell membrane and plays an important role in mitochondrial biogenesis [37,38]. ALA seems to share the same mechanisms as metformin, which can also activate AMPK in hepatocytes and skeletal muscles [39].

Moreover, within the cell ALA could carry out additional functions associated with glucose metabolism; in particular, it could influence mitochondrial biogenesis and endoplasmic reticulum (ER) activity. In fact, ALA stimulates the expression of mitochondrial markers such as TFAM, PPARɣ and PGC1α in C2C12 cells (a myoblast cell line) and the expression of gene encoding for representative antioxidant enzymes such as glutathione peroxidase (GPX1) and superoxide dismutase 1 (SOD1) [40]. In glucose-treated rats, the decrease in PPARγ protein levels caused by oxidative stress is prevented or attenuated if they are fed with ALA [41]. However, to date, the contribution of ALA-induced PPARγ expression to insulin sensitivity cannot be clearly quantified. In the endoplasmic reticulum, ALA increases the endogenous expression of DNAJB3 cochaperone, heat shock proteins (HSP25 and HSP27) in C2C12 cells [40,42]. DNAJB3 cochaperone has been associated with reduced metabolic stress, improved insulin signalling and glucose uptake in previous in vitro and in vivo studies (3T3-L1 adipocytes and human obese subjects) [43,44]. However, it is unclear whether DNAJB3 exerts a direct effect on ER stress or acts indirectly through other pathways. Similarly, low HSP expression could play a critical role in the induction of insulin resistance and diabetes [45]. ALA’s pro-oxidant properties also include the induction of HSP and the activation of PI3K.

Thus, the effect of ALA on GLUT4 is certainly the major determinant of the increase in glucose uptake, making ALA an insulin mimetic agent. However, it is still unclear how ALA interacts with the insulin receptor and insulin receptor cascade. It is possible that ancillary effects of ALA on mitochondria and the endoplasmic reticulum further improve insulin sensitivity. The effects of ALA on glucose metabolism seem not to be limited to a peripheral insulin mimetic effect, as some evidence suggests that it also acts on beta-cells.

### 4.2. Effect of ALA on Pancreatic Beta-Cells

Several studies over the last two decades have explored changes in insulin secretion during ALA treatment. Most of these studies investigated the effect of ALA in vitro in the presence of substances toxic for beta-cell function, such as oleic acid (OA), which can interfere with mitochondria and insulin secretion [46] and 2-deoxy-D-ribose (dRib), a strongly reducing sugar which induces apoptosis and oxidative stress in beta-cell lines [47]. Pre-treatment with ALA (10 mM) prevented OA-induced decrease in insulin secretion in MIN6 cells [48] and reversed low mRNA insulin levels in a dose-dependent manner in dRib [49] stimulated HIT-T15 cells. These studies suggest that in beta-cells ALA acts as an antidote to toxic substances which reduce insulin secretion.

This hypothesis has also been confirmed in studies in vivo. In T2D rats fed with fructose, ALA did not affect insulin sensitivity but induced greater insulin excursions after glucose administration [50]. In a more recent study, the co-administration of lypo-polisaccharide S (LPS) (an inducer of chronic subacute hepatic inflammation, which can also impair pancreatic insulin secretion) [51] and ALA produced no changes in insulin sensitivity [52], assessed by euglycemic hyper-insulinemic clamp [53], but determined a restoration of first-phase and second-phase insulin secretion [54] (assessed by hyperglycaemic clamp), compared to rats treated with LPS alone. In aged rat islets, ALA significantly increased insulin secretion and decreased reactive oxygen species [55]. We can consider the latter as a model of oxidative stress induced cell damage, even though it differs from the other studies due to the absence of exogenous toxic substances.

In relation to the structure of pancreatic beta-cells, further studies suggest that ALA could exert a protective effect against pancreatic injury induced by Ciclosporin A [56] and Valproic acid [57]. Ciclosporin A is a potent immunosuppressive drug, listed among the possible etiologic causes of post-transplant diabetes mellitus (PTDM). In this specific context, ALA seems to protect islets from atrophy and increase insulin-immunoreactive granules.

IL-1β is produced by activated macrophages and could be the responsible for early beta-cell injury in type 1 diabetes. When ALA was administered to isolated islets from NOD mice treated with IL-1β, insulin secretion returned to normal. However, this effect was dose-dependent since although a 10^−9^ M lipoic acid concentration was effective, 10^−6^ M and 10^−12^ M concentrations produced no effects. Interestingly, when the 10^−9^ mM lipoic acid concentration was tested alone without the addition of IL-1β,insulin secretion was not restored, but, in fact, decreased significantly (68%) compared to the control condition [58]. The latter observation suggests that the effect of ALA on insulin secretion can change in relation to more complex intracellular dynamics [59].

The administration of ALA alone leads to similar results in vitro, as shown in two different clonal beta-cell types (HIT-T15 cells [60] and in MIN6 cells [3]), as ALA inhibited glucose-induced insulin secretion in both. While in HIT-T15 cells this finding was also accompanied by a reduction in beta-cell apoptosis, in MIN6 cells this effect was associated with AMPK α-subunit phosphorylation and activation and was compared to other well-known AMPK activators, such as AICAR and metformin. In high glucose conditions all these compounds, administered chronically, decreased insulin secretion and, moreover, ALA treatment produced an acute decrease in glucose-stimulated insulin secretion (GSIS) while AICAR and metformin did not. Thus, ALA’s effects on beta-cell AMPK parallels its effects in skeletal muscle, even though with opposite effects, as it increases insulin sensitivity while decreasing insulin secretion. The administration of ALA alone, therefore, seems to worsen insulin secretion, at least in beta-cell lines. However, we could consider the overall effect as protective, because improved insulin sensitivity accompanied by a smaller demand for insulin production may protect beta-cells from exhaustion [7,61,62].

In addition, a recent study by Azzam et al. suggests that lipoic acid and ascorbic acid—among other antioxidant compounds—have a significant inhibitory activity on the fibril aggregation of amylin [63], which is physiologically co-localized in granules with insulin in beta-cells. Thus, both ALA and ascorbic acid could play a protective role in β-cell overload, which has been correlated with plasma amylin levels in T2D [64]. Furthermore, ALA successfully counteracts other mechanisms of amyloidosis such as the formation of β-amyloid fibrils [65].

The effects of ALA on insulin secretion, therefore, vary according to the oxidative balance of the cell or the presence of other substances. In vitro and in vivo models suggest a role in the prevention or attenuation of the pathogenetic mechanisms that lead to type 1 and type 2 diabetes, diabetes associated with liver diseases and post-transplant diabetes.

### 4.3. ALA for the Treatment of Diabetes

Diabetes is a chronic disease characterized by chronic hyperglycaemia. While Type 1 diabetes is the result of a reduced or absent insulin secretion, Type 2 diabetes is characterized by both insulin resistance and reduced insulin secretion in different proportions [66].

Given the positive effects of ALA on insulin sensitivity and the protective effects on damaged beta-cells, we would expect some therapeutic efficacy in the management of diabetes.

A metanalysis [67] of six studies including uncomplicated T2D patients, showed no differences in terms of HbA1c between the control group and the group treated with ALA. Most of the patients enrolled were taking other antidiabetic drugs like metformin, sulphonyl-ureas and insulin in different combinations. None of these studies specifically included drug-naïve patients and only one study showed an improvement in HbA1c and other metabolic parameters in patients with complicated T2D treated with ALA [68]. The latter study evaluated 90 elderly type 2 diabetic patients with acute cerebral infarction randomized to receive ALA or vitamin C for 3 weeks plus insulin therapy. With the same insulin doses, FPG, 2HPG and HbA1c decreased after treatment in both groups, but the changes in the ALA group were greater compared with the control group.

Another metanalysis [69] reported a significant decrease in FPG, HbA1c, insulin concentrations, HOMA-IR, triglycerides and LDL cholesterol in individuals with metabolic diseases treated with ALA. In this case, the overall population analysed included subjects who were only overweight or had diabetes, metabolic syndrome, or polycystic ovarian syndrome. Half of the total number of eligible studies (i.e., 12 papers) examined the effects of ALA on glucose control and/or lipid profiles in T2D patients, only a few evaluated changes in HbA1c and the rest evaluated only FPG or HOMA-IR.

Therefore, despite the promising results in animal and in vitro models, treatment with ALA has had controversial and mostly disappointing results in studies on the treatment of diabetes in humans.

A recent study [70], not included in the previous metanalysis, confirms these data in glibenclamide/metformin-treated patients as no statistically significant differences were found in terms of HbA1c and glutathione peroxidase (Gpx) between ALA group and placebo group. The authors, however, argue that the negative results are due to low doses of ALA.A dose dependent effect could also be assumed in the study by Porasuphatana [71], which included metformin and sulphonyl-ureas-treated patients randomly divided into five groups to receive either placebo or four different doses of ALA. The changes between pre- and post-treatment in each group failed to reach a statistical significance due to the small sample size. Only when all patients in the ALA groups were pooled and compared to the placebo group was there a significant difference in terms of HbA1c and a significant correlation between ALA doses and HbA1c. Studies evaluating the effect of ALA on HbA1c in humans are listed in Table 1.

Thus, further studies with high doses of ALA are needed and the drug should be tested as add-on therapy to newer and safer antidiabetic drugs such as SGLT2 inhibitors and GLP-1 receptor agonists.

Although the effects on HbA1c are limited, the studies carried out with hyper-insulinemic euglycemic clamp in T2D showed increased insulin sensitivity both after acute parenteral administration [72] and after one-month oral treatment [73,74]. In particular, in the study by Kamenova [74], glucose disposal rate after treatment did not differ statistically from that of a control group of normal glucose-tolerant subjects. This discrepancy between ALA’s effect on insulin sensitivity and glycaemic control is not surprising. Insulin resistance and glucose control are different parameters and insulin resistance counts only partially towards the pathogenesis of diabetes and its manifestations. Thus, ALA could be effective in those phases in which the insulin sensitivity defect is predominant but not when beta-cell failure has already occurred. This would also explain the difference in results between the two previously mentioned meta-analyses, as the latter probably included a higher number of insulin resistant patients with normal insulin secretion. This ambivalent effect has also been observed in prediabetes, which is also characterized by a beta-cell defect [75]. In this setting, ALA had no effect on body weight, percentage body fat, blood pressure and glucose level compared to placebo, but decreased plasma insulin and HOMA-IR [76]. In women with gestational diabetes, however, the administration of ALA 100–300 mg per day led to a reduction in fasting glucose levels, although no insulin data were available [77,78]. Studies evaluating the effect of ALA on insulin sensitivity and secretion in human are listed respectively in Table 2 and Table 3.

**Table 1:** Studies on ALA’s effects on HbA1c *.

	Country/Population	Intervention	Duration	Other Antidiabetic Drugs	Results
[6] Ziegler et al., 1997	Germany/T2D	ALA 800 mg or placebo (oral administration) per day	4 months	14 patients on oral antidiabetics while 47 patients on insulin therapy	No HbA1c differences between ALA and placebo
[79] Heinisch et al., 2010	Austria/T2D	ALA 600 mg or placebo (oral administration) per day	3 weeks	23 patients on oral antidiabetics (metformin, glitazones and sulphonylureas), 5 patients on insulin and one patient no therapy	No HbA1c differences between ALA and placebo
[80] Hegazy et al., 2013	Egypt/T1D	ALA 600 mg or placebo (oral administration) per day	4 months	Insulin therapy	No HbA1c differences between ALA and placebo
[71] Porasuphatana et al., 2011	Thailand/T2D	ALA 300 mg, 600 mg, 900 mg, 1200 mg or placebo (oral administration) per day	6 months	Metformin and/or sulphonylureas	No differences between placebo and other groups, HbA1c significantly decreased in all treatment groups compared to placebo
[81] Udupa et al., 2012	India/T2D	ALA 300 mg or Vitamin E or Omega-3 or placebo (oral administration) per day	3 months	Metformin plus Glimepiride	No HbA1c differences between ALA and placebo
[82] Huang et al., 2013	China/T2D	ALA 600 mg or placebo (intravenous infusion) per day	3 months	Short term continuous subcutaneous insulin infusion	No HbA1c differences between ALA and placebo
[68] Zhao et al., 2014	China/T2D complicated by acute cerebral infarction	ALA 600 mg or placebo (intravenous infusion) per day	1 month	Insulin therapy	ALA significantly decreased HbA1c compared to placebo
[70] Mendoza-Núñez et al., 2019	Mexico/T2D	ALA 600 mg or placebo (oral administration) per day	6 months	Metformin/glibenclamide	No HbA1c differences between ALA and placebo

* Glycated Haemoglobin (HbA1c) estimates blood glucose levels of an individual over the last 3 months.

Therefore, alpha-lipoic acid could be considered in the prevention of glucose metabolism alterations rather than in their treatment. In the next section, we will analyse the effects of ALA on syndromes characterized by alteration of insulin levels and signalling without impaired glucose levels.

### 4.4. ALA for Treatment of PCOS and Conditions of Insulin Resistance

Polycystic ovary syndrome is characterized by hyperandrogenism and ovarian dysfunction [85]. It is also well known that these pathological findings often overlap with obesity (32% of PCO patients in USA) [86] and insulin resistance (up to 75% according to some authors) [87].Moreover, insulin resistance is an important contributor to the development of T2D [88] and its prevalence is also increased in PCOS [10,89]. A number of studies have performed a detailed analysis of the metabolic profile of obese PCO patients, showing that these subjects have reduced insulin sensitivity, but increased insulin secretion compared to obese adolescents without PCOS, as both first-phase and second-phase insulin secretion were higher compared to controls [90]. Therefore, the prevalence of a beta-cell defect appears to be low in these patients.

Of all the studies exploring the effect of ALA treatment in PCOS, only one performed a euglycemic hyper-insulinemic clamp, which is the gold standard for the evaluation of insulin sensitivity [91], showing a significant improvement in insulin resistance, without changes in body weight, after ALA treatment [83].

Other studies report an improvement in insulin sensitivity expressed by HOMA-I or maximal insulin response during OGTT. In a group of obese PCOS patients, ALA administration significantly decreased insulin, glucose, BMI and HOMA index after 12 weeks compared to baseline values. The degree of improvement was higher especially in those with a family history for diabetes [9]. This could suggest a protection against diabetes onset in subjects at risk of diabetes. In another study by the same group [92], overweight patients were treated withmyo-inositol1 g/die, or alpha-lipoic-acid 400 mg/die, or myo-inositol 1 gr/die + alpha-lipoic acid 400 mg/die. All treatments determined a reduction in HOMA-IR and maximal insulin response. No significant differences were found in the ALA groups in subgroups with and without T2D familiar history. ALA treatment, however, does not affect sexual hormone parameters while myo-inositol does.

The combination of alpha-lipoic acid and inositol has been evaluated in several studies and beneficial effects have been reported despite different dosages and length of observation [93,94,95]. HOMA-IR decreased especially in the studies that documented a condition of greater insulin resistance at baseline. For example, in the study by Fruzzetti et al. [93], HOMA-I significantly decreased only in insulin resistant subjects, while no changes were observed in the group of subjects without insulin resistance.

In addition, the combination of ALA and inositol also determined a decrease in menstrual abnormalities [94,95]. Cycle length was progressively reduced in oligomenorrheic women after 24 months of treatment [94] and there was also a significant decrease in BMI [93,94]. Hormonal parameters also improved in the study by De Cicco et al. [96] in which treatment was administered at a double dose (ALA 800 mg and myo-inositol 2000 mg). They found that androstenedione and DHEA-S significantly decreased and mean SHBG increased and AMH serum levels decreased significantly compared to baseline. Promising results were also obtained in patients undergoing in vitro fertilization [97,98]. Moreover, the association of alpha-lipoic acid, myo-inositol and metformin (1.7 g) determined a greater improvement in hyperandrogenism, BMI and HOMA index than metformin alone (3 g) [99].

Few studies have been conducted on the effects of this molecule in other states of insulin resistance. In a study by Xiao et al. [84], there were no beneficial effects on lipid-induced impairment in insulin sensitivity and secretion, evaluated with clamp techniques (hyperglycaemic clamp followed by euglycemic hyper-insulinemic clamp the same day) in overweight and obese patients without T2D.

By contrast, other studies [100,101,102] show better metabolic profiles following ALA treatment. In a cross-over, double-blind placebo-controlled study, ALA 1200 mg positively affected blood pressure, BMI, waist circumference and triglyceride levels compared to placebo [100]. Moreover, two metanalyses [101,102] have confirmed a significant—even though minimal- reduction of BMI and weight with ALA treatment. In the metanalysis by Kucukgoncu et al., overall weight loss was 1.27 kg greater with ALA treatment than in the placebo group and BMI difference was 0.40 kg/m^2^ between the two groups. In the metanalysis by Namazi et al., there was a slight effect on body weight and BMI: respectively 0.69 kg and 0.39 kg/m^2^ compared to placebo. However, the reduction in waist circumference was not significant and there was no correlation between weight loss and ALA dose. Interestingly, both metanalyses indicate that shorter treatment duration achieves greater BMI reduction than longer intervention. Therefore, we could conclude that ALA is effective for weight loss but with no certain durability of the results.

## 5. ALA and Diabetic Neuropathy

Diabetic neuropathy is a known microvascular complication of diabetes. Data on the benefits of alpha-lipoic acid for both diabetic polyneuropathy (DPN) and diabetic autonomic neuropathy (DAN) started emerging in the 1990s [103,104]. DPN is one of the main clinical presentations [105] and is characterized by “negative” symptoms such as hypoesthesia and “positive” symptoms such as paraesthesia, dysesthesia, allodynia, or pain. The latter has been identified as the major determinant of depressive symptoms in people with diabetes, as it interferes with the ability to enjoy life, with daily activities and with sleep [106,107]. Electroneurography is the main diagnostic tool, confirming the presence of DPN in symptomatic patients and highlighting alterations in peripheral nerve conduction [105]. Systematic reviews and metanalyses [108,109] have evidenced the beneficial effects of ALA in the treatment of diabetic polyneuropathy (DPN). ALA, administered at a dosage of 600 mg or more either intravenously or orally, improves the Total Symptoms Score (TSS) and Neuropathy Impairment Score (NIS) in people with DPN [108,109]. ALA was shown to improve night pain, paraesthesia, muscle atrophy and difficulty in walking in a cohort of 20 patients. Better results were obtained in patients with lower HbA1c <7% than in those with higher HbA1c >7% [110].

The effects of ALA appear not to be limited solely to the symptoms of diabetic neuropathy. Indeed, ALA can be considered as the only treatment that acts on the pathogenesis of the disease while the others—α2δ ligands, tricyclic antidepressants and opioids— are only symptomatic treatments for pain. The mechanism may be related to an improvement in nerve blood flow and distal nerve conduction mediated by the antioxidant action [111,112].A systematic review including 15 articles, examined the effect of ALA 300–600 mg i.v. per day for two to four weeks, on motor nerve conduction velocity (MNCV) and sensory nerve conduction velocity (SNCV). Both these parameters increased after ALA administration even though most of the studies included in this review were of poor methodological quality [113]. Despite these positive premises, to date, no pharmacological therapies (ALA included) have been able to modify the history of the disease or reverse it [114] and, according to the American Academy of Neurology, there are no conclusive data for the use of ALA or B vitamins in the treatment of pain [115].

The manifestations of autonomic diabetic neuropathy are very disparate since different systems can be involved. Cardiovascular autonomic neuropathy (CAN), for example, can manifest with tachycardia, reduced heart rate variability, QT lengthening, orthostatic hypotension, reverse dipping and reduced sympathetic-adrenergic response to hypoglycaemia. CAN is a risk factor for cardiovascular morbidity and all-cause mortality [116]. In the Deutsche Kardiale Autonome Neuropathie Studie [117], diabetic patients with CAN—on oral anti-hyperglycaemic therapy—treated with ALA, experienced an improvement in heart rate variability, while QTc decreased but not significantly.

As regards the use of ALA in the management of other microvascular complications of diabetes, the results are rather disappointing. In the REPITON trial [118], a daily dose of 600 mg ALA did not prevent the occurrence of clinically significant macular oedema in diabetic patients. Similarly, a recent metanalysis concluded that ALA supplementation does not improve biological indices that reflect diabetic nephropathy in humans, even though with limited evidence [119].

The possibilities for use of this molecule for glyco-metabolic pathologies are therefore ample, but attention must be paid to possible side effects.

## 6. ALA and Insulin Autoimmune Syndrome

Insulin Autoimmune Syndrome is a rare cause of endogenous hyper-insulinemic hypoglycaemia [120] It was first described in 1970 in Japan by Hirata and colleagues. Since then, several cases have been identified and nowadays IAS is reported to be the third most common cause of spontaneous hypoglycaemia in Japan [121].

IAS is characterized by the presence of a high titre of insulin autoantibodies (IAA) which bind to insulin and proinsulin. They thus interfere with insulin action, determining a transient phase of hyperglycaemia and a prolonged pancreatic secretion of insulin and C-peptide. When IAA dissociate from insulin, hypoglycaemia occurs [122]. It can be lasting and even more dangerous than that seen in patients with insulinoma.

In 2007, Uchigata et al. published a report on the first case of ALA-induced Insulin Autoimmune Syndrome [123]. To date, 49 cases can be retrieved by a comprehensive literature search [124]. The majority of cases have been reported in Japan, while in Europe most cases have been described in Italy, two by our group in 2018 [15].The pathogenesis of ALA-induced IAS has not been fully elucidated. It seems that certain individuals could be more susceptible to the development of the disease after the introduction of substances containing sulfhydryl groups, such as ALA. The presence of the Human Leukocyte Antigen HLA-DR4 [125] and, in particular, the DRB1*04:06 and DRB1*04:03 alleles (most of the European cases) are associated with an increased risk of IAS. In Japan, 97% of affected patients are HLA-DR4-positive and 43% are also DRB1*0406-positive [12].In Europe, the predominant allele is *0403. This difference is also linked to the different prevalence of these two alleles: in Japan the frequency of the allele*04:06 is reported to range between 5.3% and 13.2% while in Europe it is between 0.1 and 1%. Conversely the frequency of allele*0403 is 1.6–12.3% in Japan while its prevalence is 0.4% to 3.9% in Europe [126]. Before ALA-induced IAS was discovered, other sulfhydryl compounds, such as methimazole, had been associated with IAS [127]. These compounds, due to the reducing properties conferred by sulfhydryl groups, could linearize insulin α chain by cleaving its disulphide bond. In this way hidden fragments of human insulin would be exposed and recognised by T lymphocytes, triggering an immune response and eventually antibody production. Matsushita et al. [128] detected the insulin fragment (TSICSLYQLE) with the highest affinity to DRB1*0406. Conversely the allele DRB1*0405, which differs from the latter for some amino acid residues, exhibits an IC_50_ value 44 times higher [128]. HLA- DRB1*0403 is closely related to HLA-DRB1*04:06 and it has been suggested that HLA-DRB1*04:03 is an evolutionary predecessor of HLA-DRB1*04:06 [129]. Interestingly, allele DRB1*0403 is protective against type 1 diabetes [130] and primary adrenal insufficiency [131] while both DRB1*0403 and DRB1*0406 could predispose to pemphigus [132]. To date, the exact interactions of ALA and methimazole with insulin in IAS are unclear, but it can be asserted that both a reducing ambient and a genetic predisposition seem to be necessary for the development of the disease.

In case reports, the time to onset of IAS after taking ALA ranges from 1 week to 4 months (7–120 days) with no obvious association between dose and time to onset. Hypoglycaemic episodes are generally postprandial, but they have also been reported in the fasting state. Insulin levels are very high and C-peptide levels are normal or high. After PEG precipitation, recovery of insulin is low (5–10%) compared to control samples (70%) [13]. The reason for high C-peptide levels is still controversial. C-peptide may cross-react with the same IAA and may also be “incorrectly” reported as “free” C-peptide by some immunoassays [122]. Furthermore, the initial transient hyperglycaemia—due to insulin binding to antibodies—in a usually euglycemic subject could stimulate beta-cells and determine C-peptide release. The severity of hypoglycaemia is linked to antibody titres and their affinity: low affinity antibodies are more likely to cause hypoglycaemia [122,133]. However, in some clinical situations IAA are not pathogenetic. In a study on T2D patients, insulin antibodies were detected in 48 of 118 patients (40.7%) on insulin therapy and were unexpectedly found in seven of 263 insulin naïve patients (2.7%) [134].

IAS usually resolves once the trigger is removed. There is no agreement as to whether to introduce a therapy or even which therapy to use. In case reports, diazoxide, prednisone and other immunosuppressants were administered after ALA suspension. In all cases, hypoglycaemic events disappeared after a comparable period of time (one week to three months) [124]. Thus, the introduction of drugs is not mandatory, but based on the severity of symptoms and the patient’s general clinical condition. However, the positioning of a flash glucose monitoring (FGM) device could be a reasonable option to monitor the possible persistence of hypoglycaemic episodes over time [135]. In only one case report ALA was wrongly re-administered after disease remission in a South Korean 67-year-old woman with type 2 diabetes and neuropathy. She underwent two ALA re-challenges after the first administration and in both cases developed Insulin Autoimmune Syndrome [136].

Recently the European Commission has asked the European Food Safety Authority (EFSA) to review existing data on the possible link between ALA administration and IAS and provide advice on the dietary intake of foods supplemented with alpha-lipoic acid. A panel published by EFSA in April 2021 [124] concluded that the addition of ALA to foods is likely to lead to an increased risk of development of IAS in individuals with certain genetic polymorphisms. On the other hand, no link has been found between the intake of foods naturally containing ALA with IAS. Furthermore, an ALA dose below which IAS is not expected to occur cannot be derived based on the available data. This is probably due to the autoimmune pathogenesis of the disease.

Therefore, we can conclude that the safe daily administration of ALA would require the analysis of HLA, although this is rarely feasible in clinical practice. An accurate evaluation of the medical history of the patient could help identify any pathologies sharing the same HLA predisposing alleles of ALA-induced IAS, such as pemphigus, thus avoiding the introduction of the drug.

**Figure 1:**
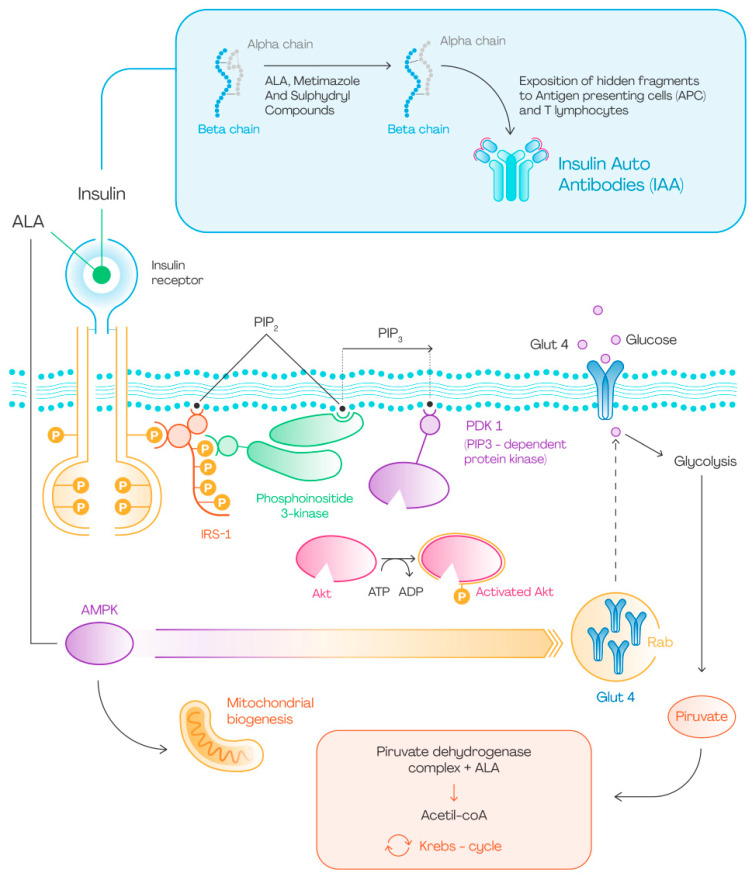
ALA and glucose metabolism.

This Figure 1 summarizes the main effects of ALA on glucose metabolism: it can activate the insulin receptor by extracellular binding, but it can also cross the cell membrane and activate AMPK. The latter interaction is responsible for GLUT4 expression on cell surface and increased glucose uptake. This promotes glucose entry into the cell and glycolysis. ALA then contributes to the beginning of Krebs cycle through its interaction with pyruvate dehydrogenase. On the other hand, ALA could modify insulin structure through the cleavage of disulphide bond and the subsequent exposition of hidden fragments to immune system. In this way lymphatic organs produce IAA which bind insulin and proinsulin and determine IAS.

## 7. Conclusions

Alpha-lipoic acid has pleiotropic effects on glucose metabolism (Figure 1), many of which are still under investigation. This compound is widely prescribed for the treatment of insulin resistant states, such as polycystic ovary syndrome and for diabetic neuropathy, due to the amount of supporting evidence.

Blood glucose is the result of many sophisticated intra- and extra-cellular processes on which ALA seems to have only a partial impact. We have seen how the effect on insulin secretion primarily depends on the degree of cellular oxidative balance [55], but that this molecule can have only a limited effect on beta-cell metabolism tout court [67]. To date, the evidence on ALA’s validity as an antihyperglycemic drug is rather disappointing, but there are few human studies on the addition of alpha-lipoic acid to more recent therapies such as SGLT2i and GLP1-RA. Further studies are needed to determine its possible role in the treatment of diabetes, identifying any sub-categories of diabetic patients who could benefit from this type of therapy [137].

However, it cannot be excluded that alpha-lipoic acid could be used to prevent rather than treat the alteration in insulin sensitivity that may precede the development of diabetes by years. The studies on ALA in PCOS seem to confirm this hypothesis. For example, overweight and obese PCO patients are characterized by increased insulin secretion and reduced insulin sensitivity [90] but have an increased risk of developing diabetes [88]. Family history is known to be a risk factor for its development and ALA has been shown to improve insulin sensitivity and reduce blood glycaemia particularly in those patients with diabetic relatives [9]. Another area where ALA treatment has become consolidated is diabetic neuropathy, due to its beneficial effects both on the pathogenesis of the disease and on pain control [113]. However, curative treatments for this pathology are still lacking and the response rates to this and other treatments are often incomplete [115].

Considering the effects of this molecule on glucose metabolism, we cannot disregard its possible interference with the immune system leading to severe hypoglycaemia. In recent years there have been many reports of ALA-induced Insulin Autoimmune Syndrome, which, however, seems to occur only in genetically predisposed individuals [124]. The exact interactions of ALA with insulin in this disease are still unclear. It probably reduces disulphide bonds of insulin α chain displaying usually hidden amino acid residues to the immune system, leading to the production of autoantibodies [128]. Great attention must be paid to the possible onset of insulin autoimmune syndrome. The analysis of HLA would make the introduction of the drug safe, but this is rarely feasible in clinical practice. When this test is not available, we believe that this molecule should be prescribed with great care particularly in pregnancy and in fragile subjects in which a hypoglycaemic crisis could have particularly serious consequences. We consider it reasonable to discourage its use in patients with a history of pemphigus. It is also important that physicians become familiar with the early symptoms and signs of this pathology in order to avoid the onset of the most severe forms. Any hypoglycaemic event in a person taking alpha-lipoic acid must lead to suspicion of IAS and to immediate discontinuation of therapy.

## Figures and Tables

**Table 2:** Studies on ALA’s effect on insulin sensitivity (M and insulin sensitivity indexes) **.

[72] Jacob et al., 1996	Germany/T2D	ALA 100 mg/500 NaCl (intravenous infusion) or placebo	During glucose clamp study	Oral antihyperglycemic therapy	Mean insulin-mediated glucose disposal increased by about 50% in ALA group (from 3.76 mL/kg/min to 5.82 mL/kg/min, *p* < 0.05), no significant change in control group (from 3.57 mL/kg/min to 3.91 mL/kg/min)
[73] Jacob et al., 1999	Germany/T2D (About 20% of the patients exhibited diabetic complications)	ALA 600 mg, 1200 mg, 1800 mg or placebo (oral administration) per day	1 month	Oral antihyperglycemic therapy	No insulin-mediated glucose disposal differences between placebo and other groups. Insulin sensitivity significantly increased in all the active groups pooled together (+27%, *p* < 0.01) compared to placebo
[74] Kamenova et al., 2006	Greece/T2D	ALA 1200 mg (oral administration) per day. A group of normo-tolerant subjects served as control	One month	Metformin (850 mg once daily to three times daily)	Mean insulin-mediated glucose disposal significantly increased in ALA group (from 3.202 to 5.951 mg/kg/min, *p* < 0.01). and after treatment was not statistically different from control group.
[83] Masharani et al., 2010	USA/PCOS with oligomenorrhoea without hyperandrogenism	Controlled-release ALA 1200 mg per day (No control groups)	4 months	Oral contraceptives and/or spironolactone	Mean insulin-mediated glucose disposal significantly increased (from 9.7 ± 1.3 mg/min/kg/mU to 11.1 ± 1.7 mg/min/kg/mU after treatment). Two subjects not on oral contraceptives experienced a doubling of periods respect to four months prior to study entry.
[84] Xiao et al., 2011	Canada/Overweight and obese patients without history of diabetes	Patients were treated in random order with oral placebo followed by NaCl infusion (SAL), oral placebo followed by intralipid infusion (IH), oral ALA 1800 mg per day followed by NaCl infusion (ALA) and oral ALA 1800 mg per day followed by intralipid infusion (ALA + IH)	2 weeks each phase	No medications	Insulin sensitivity index and disposition were 19 and 25% lower in IH and IH + ALA, respectively, vs. SAL (*p* < 0.05), indicating lipid-inducedinsulin resistance. Insulin sensitivity in ALA was similar to SAL.

** Insulin-mediated glucose disposal (M) (mg/kg/min), insulin sensitivity index (l^2^ × kg^−1^ × min ^−1^ × pmol^−1^) and disposition index (l^2^× kg^−1^ x min^−2^) are obtained from height, weight and glucose infusion rate (GIR) during the last 30–60 min of euglycemic hyper-insulinemic clamp.

**Table 3:** Studies on ALA’s effect on insulin secretion (ISR) ***.

[84] Xiao et al., 2011	Canada/Overweight and obese patients without history of diabetes	Patients were treated in random order with: oral placebo followed by NaCl infusion (SAL), oral placebo followed by intralipid infusion (IH), oral ALA 1800 mg per day followed by NaCl infusion (ALA) and oral ALA 1800 mg per day followed by intralipid infusion (ALA + IH)	2 weeks each phase	No medications	Insulin secretion rate was similar between treatment groups

*** Insulin secretion rate (ISR) (pmol/min) is derived from deconvolution of C-peptide concentrations measured during hyperglycaemic clamp.

## Abbreviations

ALA	alpha-lipoic acid
DHLA	dihydro-lipoic acid
T1D	type 1 diabetes
T2D	type 2 diabetes
MS	metabolic syndrome
PCOS	polycystic ovary syndrome
IAS	insulin autoimmune syndrome
IAA	insulin autoantibodies
IRS-1	insulin receptor substrate—1
IR	insulin receptor
HSP	heat shock proteins
PTDM	post-transplant diabetes mellitus
DPN	diabetic polyneuropathy
DAN	diabetic autonomic neuropathy
NIS	neuropathy impairment score
TSS	total symptoms score
CAN	cardiovascular autonomic neuropathy
FGM	flash glucose monitoring
